# Molecular dynamics simulations of PfAQP from the malarial parasite *Plasmodium falciparum*

**DOI:** 10.3892/mmr.2012.822

**Published:** 2012-03-05

**Authors:** YUBAO CUI, DAVID A. BASTIEN

**Affiliations:** 1 Department of Laboratory Medicine, Yancheng Health Vocational and Technical College, Yancheng, Jiangsu 224006, P.R. China; 2 Department of Physics, University of Texas at San Antonio, San Antonio, TX 78249, USA

**Keywords:** PfAQP, molecular dynamics simulations, tetramer, Asn-Leu-Ala, Asn-Pro-Ser

## Abstract

Aquaporins (AQPs) are widely distributed in all kingdoms of life and act as facilitators in the transport of water and other small solutes through cell membranes. Since the plasmodial and human AQPs are different in their primary and secondary structure, an intervention targeting plasmodial AQP without affecting human AQPs is discussed to identify an attractive novel target against malaria. Therefore, it is crucial to understand the action mechanisms of these plasmodial AQPs. To explore the progression of the plasmodial real AQPs *in vivo* at work, a molecular dynamic simulation system was successfully developed for a PfAQP tetramer *in silico*. The results showed that the transporting work was not synchronous in the four channels at the same time, and that it was different at different times in the same channel. The hole sizes varied in different channels with time. The structure analysis showed that both hydrophobic and hydrophilic residues composed the inner surface of the channels, and the asparagines Asn-193 and Asn-70 assembled into two motifs of NLA and NPS in the center of the channel in place of the signature motifs of NPA in other AQPs. In brief, we successfully developed an equilibrated PfAQP-lipid system by molecular dynamics simulations, and investigated the structure of the PfAQP channel, which should aid our understanding of the AQP structure and its functional implications.

## Introduction

Water is the most common substance on Earth and is indispensable to human life. It diffuses freely through biological membranes, but only at a limited rate. Since the discovery of the first aquaporin (AQP) in 1993, hundreds of AQPs have been identified in organisms from all kingdoms of life, including archaea, bacteria, yeasts, protozoa, plants, animals and humans. These AQPs have been confirmed to confer to biological membranes in which they provide a much higher water permeability when compared to the lipid bilayer (1–3). AQPs from pathogenic protozoan parasites have been identified in *Plasmodium* species, *Toxoplasma gondii*, *Trypanosoma brucei*, *Trypanosoma cruzi* and *Leishmania* species (4–8). AQPs are physiologically important in facilitating the rapid and yet highly selective flux of water and other small solutes across biological membranes. However, they are also involved in the protection of the parasites from osmotic stress during kidney passages or during transmission between humans and insects (5,6). Therefore, these protozoan AQPs are regarded as novel therapeutic targets or entry pathways for chemotherapeutic compounds (5,6).

All known AQPs are tetrameric complexes, and each monomer has a conducting channel surrounded by six trans-membrane helices (TM1-6) and five connecting loops (loop A–E). Each half contains three trans-membrane helices and one short half-helix, both half-helices meet in the middle of the membrane to form a pseudo-transmembrane span. The two loops between helices 2 (H2) and H3 (loop B), and between H5 and H6 (loop E) have a highly conserved Asn-Pro-Ala (NPA) motif, and they meet in the center of the membrane to form the channel (9,10). At present, over 10 atomic structures of AQPs have been published within the protein structure database, wherein they provide insights into the molecular mechanisms acting in AQPs. However, no real AQPs at work can be observed, including their dynamic processes. Furthermore, there is no experimental method in sufficient spatial and time resolution to monitor permeation through AQPs on a molecular level.

In the past few years, there has been an increase in research pertaining to the molecular dynamic simulations of AQPs and complement experiments. Previous studies have provided the processes of the bimolecular system at atomic resolution in order to understand the molecular mechanisms that underlie this marked efficiency and selectivity of these channels (11). For example, molecular dynamics (MD) simulations revealed that the acting mechanism was conducted by the two-stage filters based on the structures of human AQP1 and the bacterial glycerol facilitator GlpF. The first stage is located in the central part of the channel at the NPA region, and the second stage is located on the extracellular face of the channel in the aromatic/arginine (ar/R) constriction region (12,13). The ar/R constrictions of AQP1 and GlpF are located at the narrowest point of the channels, and different side chains at the ar/R constriction regions affect the polarity and diameter of the channel bottleneck (11). The ar/R region of AQP1 is regarded as a filter that allows the permeation of small polar solutes, whereas in GlpF this filter mechanism does not apply, resulting in different permeation characteristics between AQP1 and GlpF (12,13). By contrast, NPA constriction is vital for proton exclusion by a large electrostatic barrier. However, the contribution of proteins in electric fields and desolvation effects on the barrier remain controversial (11,14). In brief, MD simulations provide noteworthy insights into the water channels that possess such high selectivity and efficiency in water or glycerol passage, while simultaneously managing the exclusion of ions.

In the present study, the single protozoan AQP structure PfAQP from the malarial parasite *Plasmodium falciparum* was modeled from its Protein Data Bank (PDF) structure file, embedded in a 1-palmitoyl-2-oleoyl-sn-glycero-3-phosphatidylethanolamine (POPE) lipid bilayer, solvated in water, and then minimized and equilibrated by means of MD simulations.

## Materials and methods

### Construction of a structural model of PfAQP

The starting coordinates for the MD simulations were from the published structure of PfAQP (PDB code 3C02). The PfAQP tetramer was generated using the transformation matrices provided with the PDF file. Topology and coordinate files were generated using the visual molecular dynamics (VMD) package (15). To solvate the protein, the SOLVATE program by Grubmüller (16) was used to fill any empty space inside the pores, as well as to surround it with water.

### Placing PfAQP tetramer in a membrane

The solvated PfAQP tetramer was embedded in a POPE lipid bilayer generated with the X and Y lengths initially set to 120Ǻ using the Membrane Builder program provided within VMD (15). The constructed membrane patch and the partially solvated protein were aligned, moved and assembled properly. Subsequently, lipids overlapping the proteins were removed by means of marking the atoms of the ‘bad lipids’ in the β field of the PDB file and deleting them using VMD. After measuring the water layer using the minmax option, the system was trimmed into a rectangular water box using VMD’s solvate plugin, where the X and Y dimensions were set at slightly smaller values than the lipid patch, since non-equilibrated membranes tend to shrink. K^+^ and Cl^−^ ions at a concentration of 0.4 mmol/l were added throughout the system using VMD’s Autoionize Plugin, which transmutes water molecules into ions. The system consisted of 64,031 atoms alltogether.

### Running simulations of PfAQP

In the beginning of the simulations, the lipid tails and bulk waters of the system were minimized and equilibrated for 0.1 ns, while the protein, crystallographic waters, ions and lipid head groups were fixed. The system was then minimized and equilibrated with the protein constrained for 0.5 ns. The ‘minimization’ run with the NAMD software (17) was performed to guide the system to the nearest local energy minimum in the configuration space, and the equilibration with the protein constrained was conducted to permit lipids, water and ions to adapt to the protein in its crystal form. Subsequently, harmonic constrains placed on the protein in the second phase were released and the entire system was equilibrated for 1.2 ns. The equilibration simulations were then run for 33.97 ns.

Simulations were carried out using the NAMD2 software package (17) with the CHARMM22 (18,19) parameter set, the TIP3P water model and 2 fs time-step intervals. Simulation pressure was maintained at 1 atm using the Langevin piston method with a fixed volume. A constant simulation temperature of 310 K and periodic boundary conditions were also employed. The Langevin dynamics and the Langevin piston methods were used to keep the temperature and pressure constant. Full electrostatics was employed using the Particle Mesh Ewald (PME) method (20).

## Results

### Equilibrium molecular dynamic simulations

**Figure 1:**
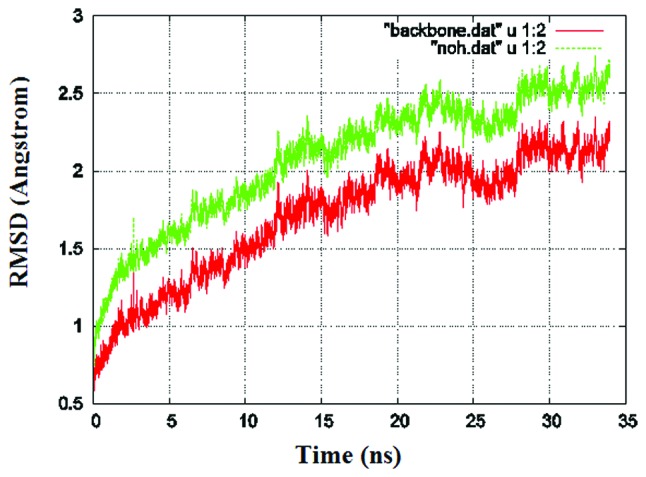
Root mean square deviation (RMSD) value of one of the monomers during the equilibrium molecular dynamics simulations. The RMSD of the protein heavy atoms (no hydrogen atoms are included) is shown in light grey, the RMSD of the protein backbone is shown in dark grey. The two RMSD values exhibited a slight change from 26.47 to 33.97 ns.

The stability of the simulation was evaluated by calculating the root mean square deviation (RMSD) value relative to the coordinates of the initial structure for the production run. Fig. 1 shows the RMSD for the whole simulation. The change of the RMSD value in the last 7.5 ns was relatively small, suggesting that the protein had reached a structural equilibrium with the lipids.

### Situations in the four channels of PfAQP tetramer at work in simulations

In simulations, the protein interacts with the lipids, and water and glycerol flow into and out of each of the four channels of the PfAQP tetramer. However, the transporting work is not synchronous in the four channels of the same PfAQP tetramer within the same period of time. Even in the same channel, the transporting work varies with time. The hole sizes were found to be different for the four channels simultaneously. Moreover, the hole sizes were different within same channel at different times. MD simulations showed the waters and glycerols molecules in each of the four channels in the initial protein-lipid complex system. The frame was shown after simulation for 26.47 ns, along with the frame after simulation for 33.97 ns, as well as the hole sizes.

### Structural analyses of the extracellular vestibule, selective filter and the conduction channel

**Figure 2:**
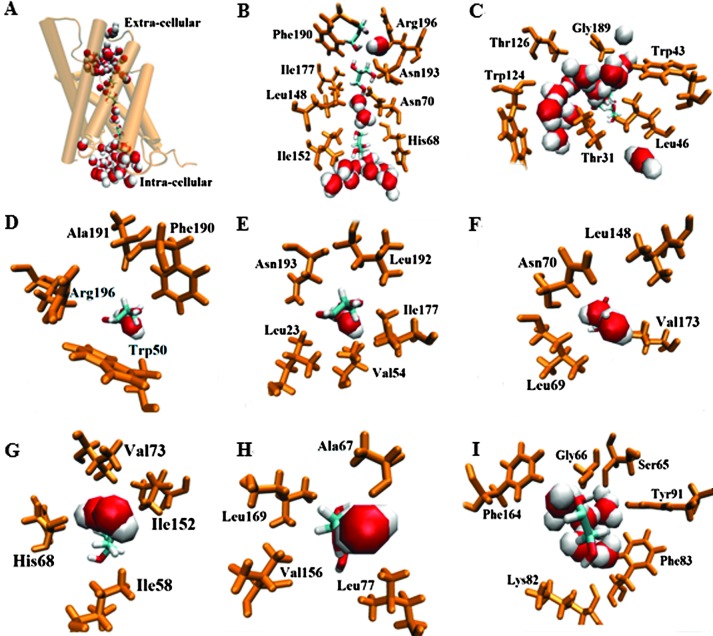
Structural analysis of the extracellular vestibule, selective filter and conduction channel is shown. The important residues are shown in the drawing method of Licorice and coloring method of orange, glycerol in the drawing method of Licorice and coloring method of name, water in the drawing method of VDW and coloring method of name. (A) Image of water and glycerol molecules transporting through the channel, obtained from the last frame of the molecular dynamic simulations of the PfAQP tetramer. The image was rendered with protein in the drawing method of Cartoon. (B) Schematic image of the protein with water and glycerol molecules through the channel. (C) The extracellular vestibule formed by Thr-31, Trp-43, Leu-46, Trp-124, Thr-126 and Gly-189. (D) The selectivity filter formed by Arg-196, Phe-190, Trp-50 and Ala-191. (E) The residue Asn-193, a member of the NLA motif residues, which interacts with water, together with residues Leu-192, Ile-177, Leu-23 and Val-54. (F) The residue Asn-70, a member of the NPS motif residues, which interacts with water in the conduction channel, together with residues Leu-148, Leu-69 and Val-173. (G) The residue His-68, which interacts with water in the conduction channel, together with Ile-58, Ile-152 and Val-73. (H) The residue Ala-67, which interacts with water in the conduction channel, together with Leu-77, Val-156 and Leu-169. (I) The residues Gly-66, Phe-83, Tyr-91, Ser-65, Phe-164 and Lys-82 form the end of the conduction channel.

The structure of the channels of PfAQP tetramer was investigated from the last frame after our simulations. Fig. 2A shows an image of the water and glycerol molecules transporting through the channel, and Fig. 2B shows the schematic image of the channels. Prior to entering the channel, the water and glycerol molecules in the simulations remained in the extracellular vestibule, which was formed by Thr-31, Trp-43, Leu-46, Trp-124, Thr-126 and Gly-189 (Fig. 2C). Due to the selectivity filter formed by Arg-196, Phe-190, Trp-50 and Ala-191 (Fig. 2D), the channel diameter was reduced to the narrowest diameter size in the channel as computed by Hole 2.0 software. Instead of the signature motifs of NPA in other AQPs, the asparagines Asn-193 and Asn-70 assembled into two motifs of NLA (Asn-Leu-Ala) and NPS (Asn-Pro-Ser) in the center of the channel (Fig. 2E and F). At a lower point in the channel towards the intracellular side, the water molecules interacted with His-68, Ile-58, Ile-152, Val-73 (Fig. 3G), Leu-77, Val-156, Leu-169 and Ala-67 (Fig. 2H). At the end of the conduction pore, the water molecules interacted with the residues Gly-66, Phe-83, Tyr-91, Ser-65, Phe-164 and Lys-82 (Fig. 2I).

## Discussion

Malaria is probably one of the oldest infectious diseases known to mankind. It is caused by the intracellular parasite *Plasmodium falciparum*, and is transmitted by the bite of an infected female Anopheles mosquito. Although the disease is curable if the infected individuals are treated rapidly and properly, the parasite rapidly develops resistance to common antimalarial drugs, such as chloroquine (21,22). To overcome the resistance, combination therapy was introduced in clinical experiments; for example, the combination of sulfadoxin and pyrimethamine has been regarded as an effective method for the treatment of malaria, but is becoming less efficacious (21,22). Artemisinin, derived from the plant *Artemisia annua*, is known to be an extremely effective anti-malarial drug and has been used in first-line treatment for the past few years. However, there have been increasing treatment failures reported recently for artemisinin combination therapy in southeastern Asia (23). Therefore, novel strategies are crucial for the treatment of malaria.

As an integral membrane protein, plasmodial AQPs play essential roles in parasites in various ways, particularly in the transportion of water and/or other permeants, such as glycerol and urea. Since the plasmodial and human AQPs are different in their primary and secondary structure, an intervention targeting plasmodial AQPs without affecting human AQPs is believed to make the parasite-host interface an attractive novel target against protozoan-caused diseases (24). The crystal atomic structure of PfAQP was resolved at 2.05 Ǻ resolution, which provided key evidence for the basis of water versus glycerol selectivity in AQP family members (25). The present study aimed to develop an MD system of a PfAQP tetramer in a lipid bilayer, and the fact that the change of the RMSD was extremely small in the last 7.5 ns demonstrates that the MD simulation was successful.

Although structures provide invaluable information regarding AQPs, only static information is provided, and no real AQPs at work can be investigated. For example, there are three glycerol and four water molecules presented in the conduction channel of the PfAQP in the initial atomic structure (25). However, our simulations showed that the pace of transporting work was different in the four channels in the same PfAQP tetramer at the same time and varied with time in the same channel, which is evident in the parasite. Of note is that no empty channel exists. There must be glycerol and water inside each of the four channels of the PfAQP tetramers in normal physiological environments. As demonstrated in our simulations, glycerol and water move in each channel with no gaps at any time. This type of arrangement was referred to as single-file permeation (10,12,13,25–28).

In the protein-lipids complex system, the lipids maintain a tight seal around the membrane proteins, so that the proton and chemical gradients are maintained between the interior and exterior of the cell. By lateral diffusion, lipids and membrane proteins are transported, where AQPs may change their shape and undergo activity-related conformational changes (29,30). In our study, the hole sizes of the same PfAQP tetramer were found to be different in the four channels at the same time, and even in the same channel at different times. This finding shows that the PfAQP protein is active. Besides the membrane moving in the protein-lipids complex system, the amino acid residues on the surface of the channels interact with the transporting water and glycerol molecules, resulting in conformational changes in PfAQP.

The glycerol and/or water permeation is passively driven by osmotic gradients, and should be regulated by structural changes of the channel. To investigate the trafficking pathway in the channels, we dissected the equilibrated PfAQP-lipid system along the XY-plane, and found that there are many amino acid residues on the inner surface of the channel, including hydrophobic residues Trp-50, Phe-190, Ile-177, Val-54, Leu-148, Val-173, Ile-58, Ile-152, Trp-43, Leu-192, Leu-23, Leu-69, Val-73, Leu-77, Val-156, Leu-169 and Tyr-91, and hydrophilic residues Gly-189, Trp-124, Arg-196, Phe-190, Ala-191, Asn-193, Asn-70, His-68, Ala-67, Gly-66, Thr-31, Thr-126, Ala-67, Phe-83, Ser-65 and Phe-164. According to the cross-sectional analysis, two sites strongly interacting with glycerol and/or water were observed, the aromatic residue/arginine (ar/R) constriction and the NPA motif, which has been found in other AQPs (10,12,13). One of these sites is referred to as the aromatic residue/arginine (ar/R) constriction, the narrowest part of the water channel, which is located close to the extracellular pore mouth. The selectivity filter of PfAQP is formed by four residues, Arg-196, Phe-190, Trp-50 and Ala-191. This is identical with the amino acid composition of *E. coli* aquaglyceroporin GlpF, which is formed by Arg-205, Phe-200, Trp-48 and Gly-191, while that of AQP1 is formed by Arg-195, Phe-56, His-180 and Cys-189 (13). Both PfAQP and GlpF have appreciable rates of glycerol condutance, whereas AQP1 is a water-specific channel. The histidine is believed to reduce the channel diameter and, together with the highly conserved arginine, provides a hydrophilic edge in juxtaposition to an aromatic residue, which are important for water-specific channels, and thereby sterically excluding the passage of glycerol (13). As in the case of GlpF, the reason that PfAQP is more hydrophobic than AQP1 is believed to be associated with the lack of the histidine in the ar/R region of GlpF.

Instead of the signature motifs of NPA in other AQPs (10,12,13,14,25–28), the asparagines Asn-193 and Asn-70 assemble into two motifs of NLA and NPS in the center of the channel. The two asparagines lie on one side of the pore, and the hydrophobic side chains of Leu-192, Ile-177, Val-54, Leu-23, and Leu-48, Val-173, Leu-69 on the other side of the pore. The residue His-68 is the following backbone carbonyl interacting with water and glycerol on the inner surface of the PfAQP channel, and the residues Ile-58, Ile-152 and Val-73 are composed of the carbonyl groups. At a lower point in the channel of PfAQP towards the intracellular side, the water molecules interact with Ala-67, and the carbonyl groups of residues Leu-77, Val-156 and Leu-169. At the end of the conduction channel, the water molecules interact with the residues Gly-66, Phe-83, Tyr-91, Ser-65, Phe-164 and Lys-82.

According to our MD simulations, prior to entering into the PfAQP channel, water and glycerol molecules remain in the extracellular vestibule consisting of Thr-31, Trp-43, Leu-46, Trp-124, Thr-126 and Gly-189. The water channel superfamily includes AQPs and aquaglyceroporins. In aquaglyceroporins, the vestibule is regarded as a place for glycerol recruitment and desolvation of solutes for transport through the channel (13,25,28).

In conclusion, a successful MD system for malaria aquaporin PfAQP was developed, which provides new insight into the dynamic process of water and glycerol through the channels. Additionally, the structure analyses of the extracellular vestibule, selective filter and conduction channel in this study should be useful in understanding the AQP structure and its functional implications.

## Abbreviations

MD: molecular dynamics
SMD: steered MD
PDB: Protein Data Bank
RMSD: root mean-squared deviation
POPE: 1-palmitoyl-2-oleoyl-sn-glycero-3-phosphatidylethanolamine
PSF: protein structure file
NPA: Asn-Pro-Ala
PME: Particle Mesh Ewald
